# Divergent serotype replacement trends and increasing diversity in pneumococcal disease in high income settings reduce the benefit of expanding vaccine valency

**DOI:** 10.1038/s41598-020-75691-5

**Published:** 2020-11-04

**Authors:** Alessandra Løchen, Nicholas J. Croucher, Roy M. Anderson

**Affiliations:** 1 Department of Infectious Disease Epidemiology, School of Public Health, Imperial College, London, UK; 2 MRC Centre for Global Infectious Disease Analysis, School of Public Health, Imperial College London, Norfolk Place, London, W2 1PG UK

**Keywords:** Bacterial infection, Epidemiology, Conjugate vaccines

## Abstract

*Streptococcus pneumoniae* is a significant cause of otitis media, pneumonia, and meningitis. Only seven of the approximately 100 serotypes were initially included in the pneumococcal polysaccharide conjugate vaccine (PCV) in 2000 before it was expanded in subsequent years. Although the invasive pneumococcal disease (IPD) incidence due to vaccine serotypes (VT) has declined, partial replacement by non-vaccine serotypes (NVT) was observed following widespread vaccine uptake. We conducted a trend analysis assembling the available evidence for PCV impact on European, North American and Australian national IPD. Significant effectiveness against VT IPD in infants was observed, although the impact on national IPD incidence varied internationally due to serotype replacement. Currently, NVT serotypes 8, 9N, 15A and 23B are increasing in the countries assessed, although a variety of other NVTs are affecting each country and age group. Despite these common emerging serotypes, there has not been a dominant IPD serotype post-vaccination as there was pre-vaccination (serotype 14) or post-PCV7 (serotype 19A), suggesting that future vaccines with additional serotypes will be less effective at targeting and reducing IPD in global populations than previous PCVs. The rise of diverse NVTs in all settings’ top-ranked IPD-causing serotypes emphasizes the urgent need for surveillance data on serotype distribution and serotype-specific invasiveness post-vaccination to facilitate decision making concerning both expanding current vaccination programmes and increasing vaccine valency.

## Introduction

The bacterial respiratory pathogen *Streptococcus pneumoniae* (the pneumococcus) accounted for 8–12% of all deaths in children between 1 and 59 months prior to the introduction of infant pneumococcal vaccination^1^. Despite extensive vaccination programmes, the bacterium still caused up to 20 cases of invasive pneumococcal disease (IPD) per 100,000 people in Europe in 2019^2^. Infants, the elderly^3, 4^, and the immuno-compromised^5, 6^ are most affected by IPD but younger adults are also at some risk. Vaccination of children has reduced IPD in both vaccinated and unvaccinated populations, as children are the main reservoir of infection, and as such the unvaccinated age groups benefit from the removal of vaccine serotypes in children^7–13^. Combined, these facts highlight the importance of vaccine development targeted at pneumococcal disease.

Almost 100 pneumococcal serotypes are known, but as a result of the complexity of conjugate vaccine manufacturing, only a fraction of these are currently included in the pneumococcal polysaccharide conjugate vaccines (PCVs). Before the first PCV was introduced, six to eleven serotypes accounted for more than 70% of IPD cases in children under 5 in Europe and North America^14^. The first commercialized PCV was a heptavalent vaccine (PCV7), which included serotypes 4, 6B, 9V, 14, 18C, 19F, and 23F. This vaccine formulation was largely based on the serotype distribution of IPD in North America^15^ and IPD incidence decreased^16^ with the elimination of vaccine serotypes (VTs). However, IPD caused by non-vaccine serotypes (NVT) has increased^17, 18^, and this serotype replacement, reduces the net effectiveness of the vaccines^19^ for both vaccinated and unvaccinated adult individuals in countries with high vaccine coverage. As such, the 10-valent PCV10 (PCV7 + 1, 5, 7F) and 13-valent PCV13 (PCV10 + 3, 6A, 19A) have been implemented with additional serotypes to further decrease the IPD burden. PCV15 and PCV20 are likely to be licensed soon^20^.

This study has four aims. First, to assess the patterns of relative serotype abundance in IPD cases before and after the introduction of PCVs in North America, Australia and selected countries in Europe (Supplementary Fig. 1). Second, to examine differences by country in these patterns in relation to the vaccines introduced in national immunisation programmes. Third, to assess the expansion of replacement serotypes in comparison with the serotypes targeted by the vaccine. The final aim is to identify potential candidates for serotypes that should be included in conjugate vaccines given increasing evidence on the invasiveness of the replacement serotypes.

## Results

### Dataset characteristics

The countries included in this study were Australia, Finland, France, Norway, and the United States of America (USA) (Table 1). The breakdown by age groups was the same in Australia, Finland and Norway, and similar to the USA. France had only two age groups, resulting from a single cut off for children ≤ 16 years and adults > 16 years. As such, we grouped older adolescents with children in all countries to maintain consistency, such that all individuals < 18 years were considered children and all those ≥ 18 years were considered adults. France included data pre-PCV7 as well as pre- and post-PCV13, whereas Australia, Norway and USA only had pre- and post-PCV13 data available. The first two years of Australian data were removed as they had limited serotype data. The USA data was taken from a published article that included only the five most common NVTs^21^. Finland, which implemented PCV10, had pre- and post-PCV10 data available. Because PCV7 was implemented only one year prior to PCV10 implementation, the Finnish pre-PCV10 data included the PCV7 implementation year. Most countries had a 2 + 1 vaccine schedule, apart from Australia (3 + 0) and the USA (3 + 1). While PCV7 was implemented in various years, the higher valent vaccines (PCV10/13) were implemented in 2010 or 2011 for all countries. Overall national IPD incidence decreased in all countries following the introduction of PCV7 and PCV10/13. The biggest decline was that in the USA after PCV7, followed by the introduction of PCV13 in Norway (Supplementary Fig. 2).

**Table 1:** Vaccination details for Australia, Finland, France, Norway and the United States, including the years of introduction, number of doses, surveillance data source, years of vaccination data available, and the serotyping performed.

	PCV introduction	Vaccine doses	Surveillance data source	Years of vaccination data available	Serotyping
Australia	PCV7: 2005 PCV10: 2009 (some jurisdictions) PCV13: 2011	3 + 0	National Notifiable Disease Surveillance System (NNDSS)	Pre-PCV7: 1999–2005 Pre-PCV13: 2006–2011 Post: 2012–2016	Quellung reaction, molecular serotyping
Finland	PCV7: 2009 PCV10: 2010	2 + 1	National Institute for Health and Welfare (THL)	Pre-PCV10: 2004–2010 Post: 2011–2016	Quellung reaction
France	PCV7: 2006 PCV13: 2010	2 + 1	Centre National de References des Pneumocoques (CNRP)	Pre-PCV7: 2001–2006 Pre-PCV13: 2007–2010 Post: 2011–2016	Fourier transformation-infrared spectroscopy, multi locus sequence typing, genomic sequencing
Norway	PCV7: 2006 PCV13: 2011	2 + 1	Meldesystem for Smittsomme Sykdommer (MSIS)	Pre-PCV13: 2006–2011 Post: 2012–2016	Quellung reaction
USA	PCV7: 2000 PCV13: 2010	3 + 1	CDC’s Emerging Infections Program/Active Bacterial Core Surveillance	Pre-PCV13: 2005–2010 Post: 2011–2013	Quellung reaction

Data from the United States was taken from a published paper^21^.

*PCV *pneumococcal conjugate vaccine.

### Temporal trends per serotype category

**Figure 1:**
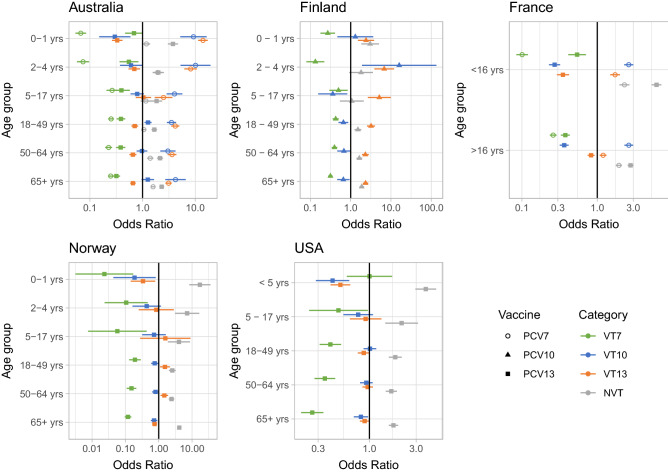
Point estimates and 95% confidence intervals (CI) of age-stratified odds ratios (OR) relating IPD caused by serotype category after vaccine implementation. Estimated as the product of the IPD cases by a serotype category post-vaccination and the IPD cases by all other serotype categories pre-vaccination divided by the product of IPD cases by the serotype category pre-vaccination and the IPD cases by all other serotype categories post-vaccination. VT7: PCV7 serotypes; VT10: 1, 5, 7F; VT13: 3, 6A, 19A; NVT: non-vaccine types.

The age group-specific odds ratio (OR) compared the difference in disease caused by one serotype category (for example, VT7) after vaccine introductions, compared to all other serotype categories and PCV eras, to determine the correlation between a vaccine employed and an increase or decrease in the proportion of disease caused by that serotype category. This shows the trends in relative incidence for different serotype categories, avoiding fluctuations in absolute prevalence that may result from non-vaccine factors. According to the OR, vaccines decreased IPD caused by the serotypes they targeted in infants in all countries, with the exception of VT10 types in one age category in Finland (Fig. 1). Decreases in VT7 IPD in all adult age categories implied a strong herd immunity effect. PCV13 consistently reduced IPD caused by VT13 in infants across countries where it was introduced, but the impact in adults was small relative to PCV7. For example, in the US and Norway, young children under 5 and the elderly were significantly less likely to get disease from VT10 and VT13 than others post-PCV13, but other age groups had no significant association between PCV13 and disease caused by VT10 and VT13. The proportion of IPD caused by NVT significantly increased across all age groups in Australia, France, Norway and the US, even in categories in which no significant decrease in vaccine types had been detected. There is evidence of herd immunity in adults caused by PCV10 administration to infants, with accompanying serotype replacement by non-VT10 serotypes across all ages. This is due to serotypes not included in PCV10 (VT13 and NVT) causing a greater proportion of disease in all age groups while non-vaccinated age groups are benefiting from vaccination with reduced proportion of disease caused by VT7 and VT10. Comparing across age groups shows herd immunity effects, despite very different absolute incidences in these different demographics. This increase in non-VT OR may represent the simple removal of VTs, or expansion of NVTs in a serotype replacement process. These two possibilities can be resolved through further analysis of IPD trends.

### Effect of vaccination on serotype diversity

**Figure 2:**
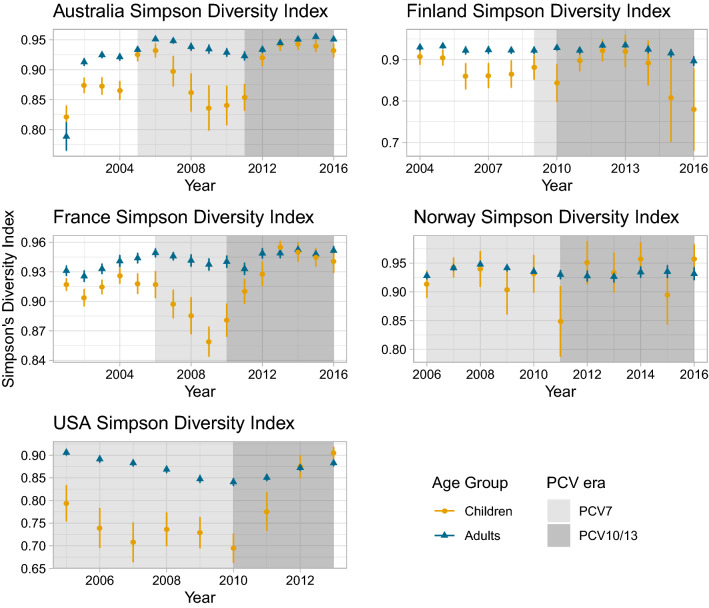
Simpson Diversity Index point estimate and 95% confidence intervals (CI) of serotypes causing IPD pre- and post-vaccination in children and adults. Children: ≤ 16 years for France, < 18 for all other countries; Adults > 16 years for France, ≥ 18 years for all other countries.

Simpson’s Diversity Index (SDI) is a measure of diversity that assesses the abundance of species in a population. In our case, estimating SDI in a population before and after vaccination can help elucidate whether vaccination changes the distribution of the serotypes causing diseases. Populations with a few dominating serotypes have a lower diversity that populations with a more even distribution of serotypes. The SDI of adult IPD remained relatively stable through the vaccination phases, indicating the diversity of serotypes causing disease stayed constant. However, significant changes were observed in children (Fig. 2). Prior to vaccination with PCV7, adult IPD isolates had a significantly higher SDI than those from children in Australia, Finland and France, implying a greater variety of serotypes caused adult IPD, which is in agreement with previous published research^15^. In Australia, France and the US, there was a fall in SDI between the introduction of PCV7 and PCV13, consistent with a few VT13 causing a large proportion of IPD. After PCV13 introduction, the elimination of these VT13 serotypes meant the SDI of disease in children increased significantly, and was no longer significantly different from that of adults. This high SDI suggests reducing infant IPD further may require targeting a wide diversity of serotypes.

**Figure 3:**
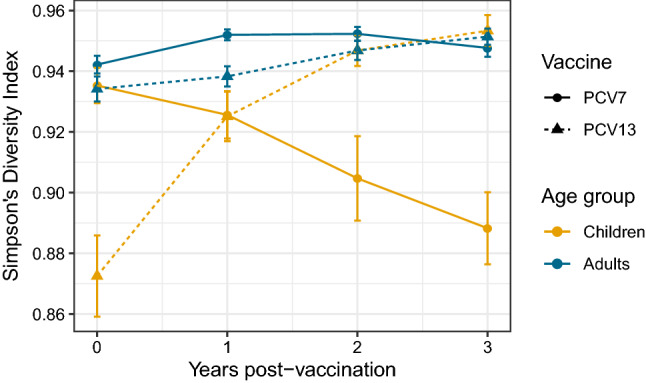
Simpson’s Diversity Index three years post-vaccination with 95% confidence intervals (CI) pooling Australia, France and Norway in children (< 18 years for Australia and Norway, ≤ 16 years for France) and adults (≥ 18 years for Australia and Norway, > 16 years for France).

Data from Australia, France and Norway were sufficiently comparable for them to be pooled over the first 4 years post-vaccination with PCV7 and PCV13 for children and adults respectively. This enables SDI to be estimated internationally. Mirroring the within-country results, there was a notable divergence between children and adults in the two post-vaccination periods (Fig. 3). SDI decreased significantly post-PCV7 in children but did not change significantly in adults, suggesting the same serotypes were dominating post-PCV7 across countries. However, post-PCV13 SDI increased dramatically in children but slowly in adults. After PCV7, diversity was considerably different between children and adults whereas post-PCV13, the diversity was not significantly different between the two age groups. This suggests the diversity of serotypes causing infant disease is now similar to that in adults, both within and between countries, making it more difficult to target with limited valency vaccines.

### Comparison of countries’ rank frequency distribution and major IPD-causing serotypes

**Figure 4:**
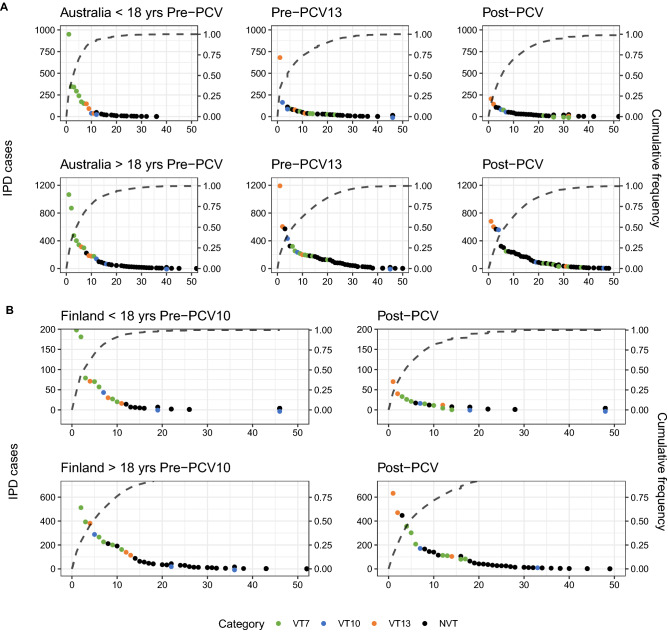

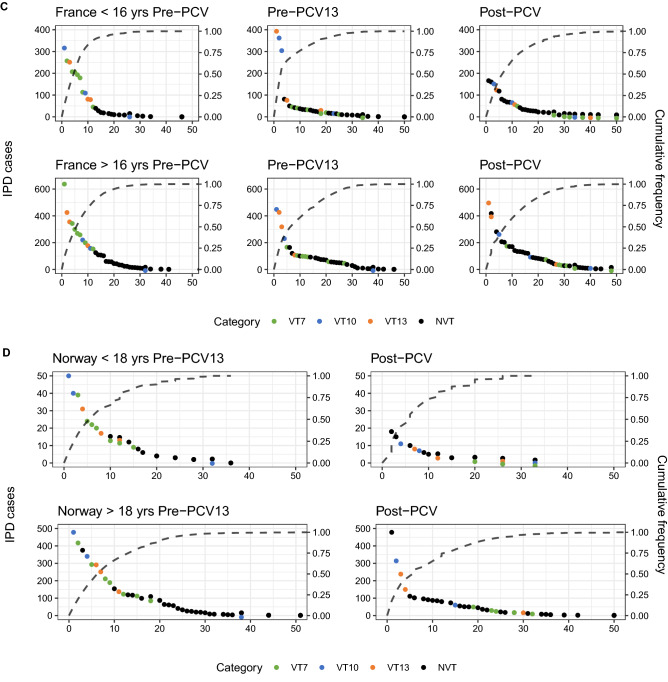

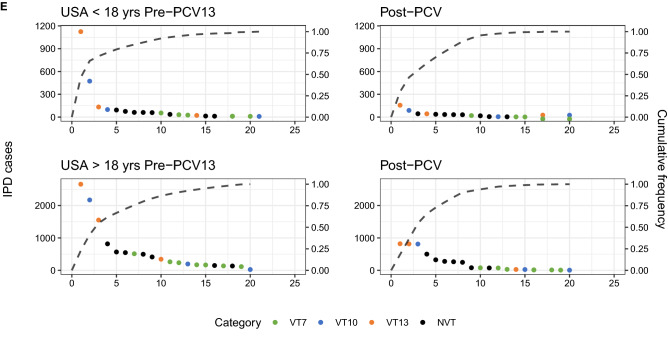
Rank frequency distribution and cumulative frequency in IPD by serotype category in (**A**) Australia, (**B**) Finland, (**C**) France, (**D**) Norway, (**E**) USA. VT7: PCV7 serotypes; VT10: 1, 5, 7F; VT13: 3, 6A, 19A; NVT: non-vaccine types.

To identify the serotypes underlying these trends, rank frequency plots, which order the top disease-causing serotypes in each PCV era by the number of disease cases caused, were combined with cumulative frequency curves of the proportion of the total IPD burden they caused (Fig. 4A–E). After vaccination, VT IPD decreased, and NVTs rose up in rankings in both age groups and countries. Each country and age group experienced, in respective pre-PCV eras, one to three serotypes causing a majority of the disease burden. Post-PCV, there were fewer ‘dominating’ serotypes, defined as those causing at least twice the IPD cases as that of the next-ranked serotype. This is also seen in the cumulative frequency curves, where the number of serotypes causing around 80% of disease in both age groups before vaccination was below ten, but increased after vaccination with PCV7. This number remained relatively stable following the introduction of the higher valency vaccines. The exception was in Norway, where there were no dominating serotypes pre- or post-PCV13 in both age groups. Serotype replacement is evident post-vaccination in all countries, which is seen independent of national surveillance agency^22^, except in the US, where the most common serotypes are in the same serotype category (VT13 and VT10) after vaccination.

**Figure 5:**
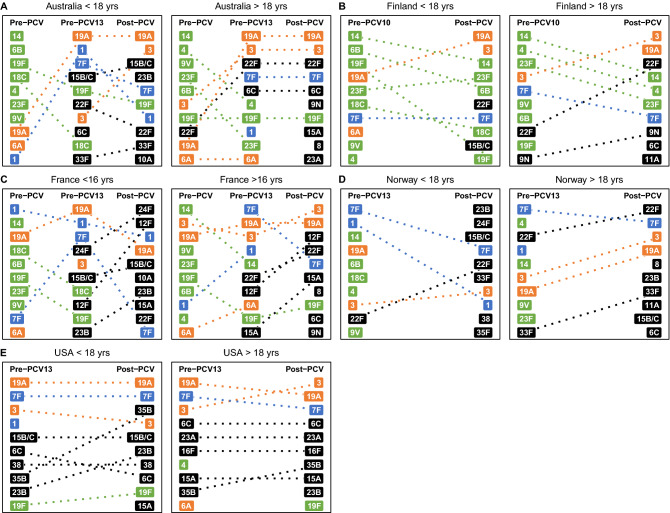
Top 10 IPD-causing serotypes pre- and post-vaccination in (**A**) Australia (**B**) Finland, (**C**) France, (**D**) Norway and (**E)** USA, with colour corresponding to serotype category. green: VT7 (PCV7 serotypes); blue: VT10 (1, 5, 7F); orange: VT13 (3, 6A, 19A); black: NVT.

The rank frequency plots suggest the most important trends in IPD can be explained by analysing the ten most prevalent serotypes. The top IPD-causing serotypes differed between children and adults and diverged post-vaccination (Fig. 5A–E, Table 2). Pre-vaccination, the countries had a similar composition of serotypes in their rankings, which consisted primarily of VTs. Serotype 14 was often dominant in both child and adult IPD. Post-vaccination with PCV7 (i.e. pre-PCV10/13), serotypes 7F and 19A dominated rankings in children, as well as serotype 3 in some settings, explaining the fall in post-PCV7 SDI. The rankings diverged post-PCV13 between children and adults, and in the same age group between countries, with NVTs dominating the rankings. Adult IPD was caused by an overlapping set of NVTs when compared with child IPD post-vaccination (Table 2). While some serotypes like 12F and 23B seemed to affect both age groups, serotypes 8 and 9N tended to appear in adult rankings more frequently and serotype 38 was more common in children. VT10 and VT13 serotypes 3, 7F and 19A persisted post-PCV10/13 in adults, with serotype 3 actually rising up the rankings in some locations. Serotype 19A still dominated the infant rankings post-PCV13 in many countries except Norway. In line with previously reported analyses^23^, there was no dominating NVT that emerged post-PCV in the same fashion that serotype 19A did post-PCV7. With the exception of the USA, the rankings were not consistent with ‘unmasking’ of NVT^24^, as NVT changed in their relative prevalence following PCV introduction. This suggests a serotype replacement process driven by the post-PCV expansion of certain NVTs.

**Table 2:** Aggregated top serotypes causing IPD in Australia, Finland, France, Norway and the United States by age group and PCV era; Vaccine categories to which serotypes belong are indicated by font: VT7 = bold, VT10 = italics, VT13 = underline, NVT = bold italics.

	Pre-PCV7	Pre-PCV10/13	Post-PCV10/13
Children	**4**, **6B**, **9 V**, **14**, **18C**, **19F**, **23F**, *1*, *7F*, 6A, 19A	**4**, 6B*, **9 V**, **14**, 18C*, **19F**, **23F**, *1*, *7F*, 3, 6A, 19A, ***6C***, ***12F***, 15C*, ***22F***, 23B*, 24F*, ***33F***, ***35B***, 38*	**6B**, **14**, **18C**, **19F**, **23F**, *1*, *7F*, 3, 19A, ***6C***, ***8***, 10A*, ***12F***, ***15A***, ***15B/C***, ***22F***, ***23B***, 24F*, ***33F***, ***35B***, 35F*, 38*
Adults	**4**, **6B**, **9 V**, **14**, **19F**, **23F**, *1*, *7F*, 3, 6A, 19A, ***22F***	**4**, **9 V**, **14**, **19F**, **23F**, *1*, *7F*, 3, 6A, 19A, ***6C***, 9 N*, ***12F***, 15A*, 16F*, ***22F***, 23A*, ***33F***, ***35B***	14, 19F, 23F, *7F*, 3, 19A, ***6C***, ***8***, 9 N*, 11A*, ***12F***, ***15A***, ***15B/C***, 16F*, ***22F***, 23A*, ***23B***, ***33F***, ***35B***

Serotypes with an asterisk indicated serotypes that do not occur in both age groups for that vaccination era. Children < 18 years in all countries except France (< 16 years), adults ≥ 18 years except France (≥ 16 years).

**Figure 6:**
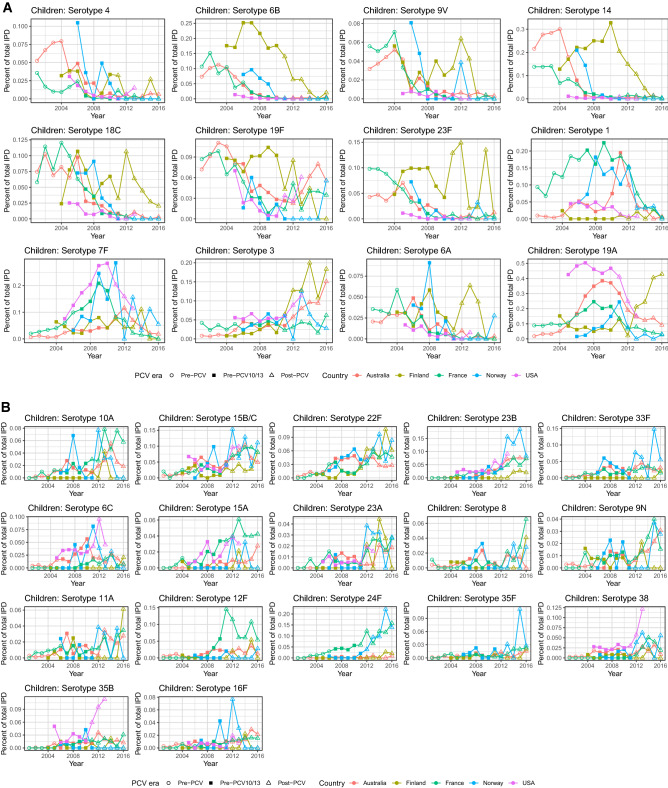

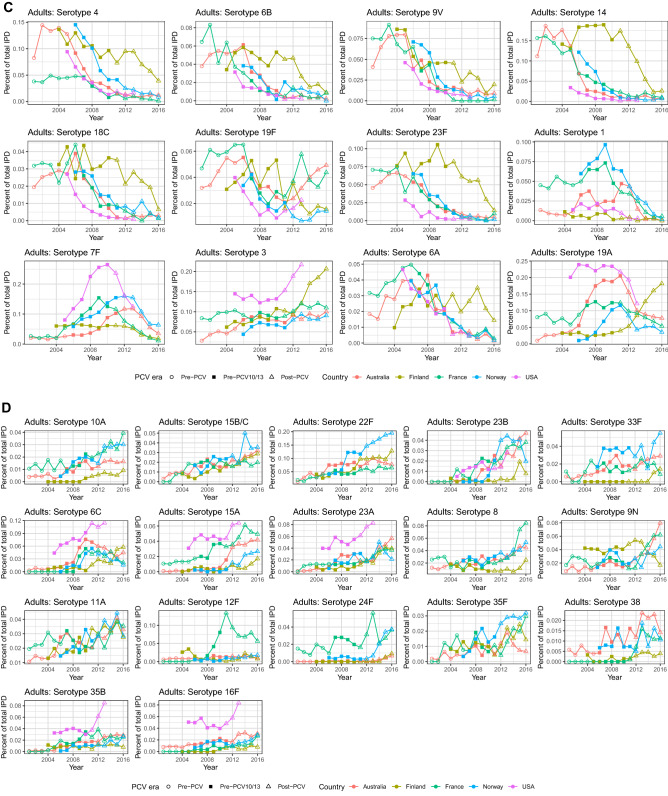
Change in percentage of national IPD caused by (**A**) VT in children, (**B**) NVT in children, (**C**) VT in adults, and (**D**) NVT in adults. Children: ≤ 16 years for France, < 18 years for all other countries. Adults: > 16 years for France, ≥ 18 years for all other countries

The fraction of total IPD caused of all these major serotypes individually over time demonstrated a similar effect (Fig. 6A–D). After any vaccine introduction, there was a steady decline in the frequency of VT7, stabilizing at levels below 5% of all IPD in most countries and all age groups. VT10 followed similar trends, although the decline in serotype 7F was still tapering off post-2012. Results are varied across settings for VT13 and NVT. VT13 serotype 6A, which was already affected by some cross-immunity with the PCV7 6B antigen, has decreased in adults and is consistently low in children. Across countries, there was a notable spike in the percentage of IPD incidence caused by serotypes 1, 7F and 19A between the introduction of PCV7 and PCV13. However, the serotype trends diverged between countries following the introduction of the expanded-valency PCVs. In the NVT serotype category, serotype 22F caused over 15% of disease in Norwegian adults post-PCV13, whereas it stayed stable around 7% in French adults. In children, serotype 22F has not increased dramatically, but instead fluctuated greatly post-PCV10/13 in most countries. Serotype 24F caused over 20% of disease in Norwegian children in 2015, and over 15% in French children in 2016, but less than 3% in Finnish children during that same time. The fraction of disease caused by serotypes 6C and 35B in American adults increased sharply post-PCV13, whereas in other countries it stayed relatively stable between 2 and 6%. The percentage of serotype 8 increased gradually in most countries’ adults, with a particularly dramatic rise in French adults.

### Serotype incidence growth rate

Effects of vaccination can be seen in the rates of incidence change of certain serotypes across countries and age groups (Supplementary Figs. 3, 4). These highlight serotypes that are rare (absent from Fig. 5) but growing due to serotype replacement, and serotypes that are common (present in Fig. 5) but declining due to vaccination or herd immunity.

#### Australia

**Figure 7:**
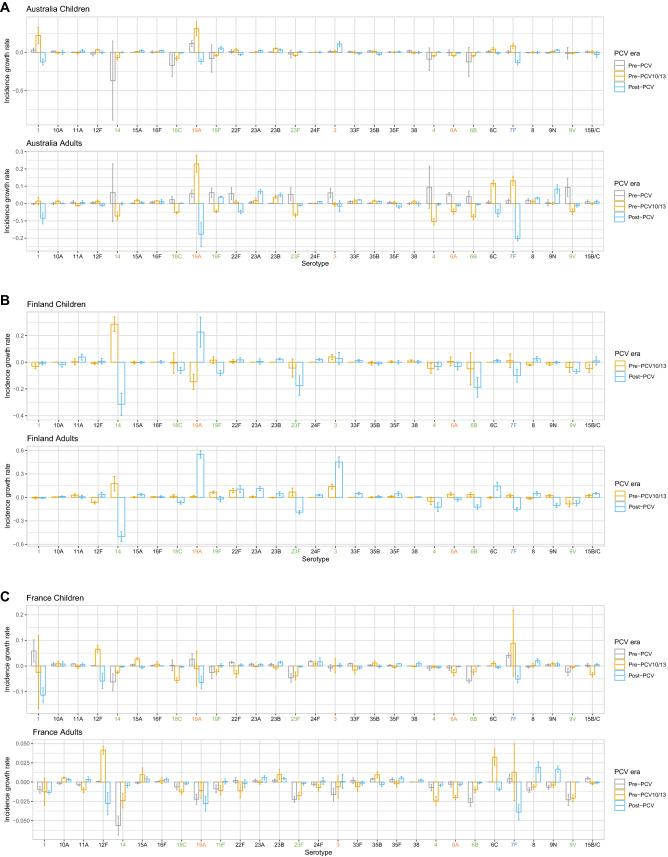

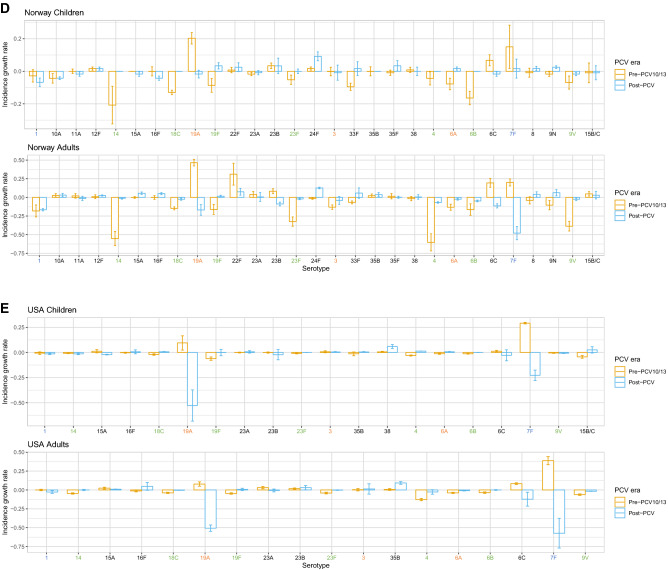
Rate of incidence growth in IPD-causing serotypes pre- and post-vaccination in (**A**) Australia, (**B**) Finland, (**C**) France, (**D**) Norway, and (**E**) USA. The x-axis labels are coloured by serotype category: green: VT7 (PCV7 serotypes); blue: VT10 (1, 5, 7F); orange: VT13 (3, 6A, 19A); black: NVT (non-vaccine types). Children: ≤ 16 years for France, < 18 years for all other countries. Adults: > 16 years for France, ≥ 18 years for all other countries.

In Australia, VT7 were not significantly changing in children in incidence pre-PCV (except 18C) but were increasing significantly in adults (except 4 and 14). Post-PCV7, VT7 decreased in incidence, whereas VT10 and VT13 serotypes (except serotype 3 in children) were, as expected, increasing pre-PCV7 and PCV13 but decreasing post-PCV13 (Fig. 7A). Children and adults had similar incidence growth rates. Serotypes that were both common (Fig. 5A) and growing were 8, 9N and 22F (adults), and 23A and 23B (both age groups). Despite its persistent presence on the rank list post-vaccination, serotype 7F was decreasing substantially in both age groups post-PCV13, as was serotype 6C in adults. From these results, we see that post-PCV13 there has not been a sharp increase in any serotype as there was pre-PCV13 with serotype 19A.

#### Finland

Incidence of VT7 and VT10 in Finland decreased post-PCV10 in both age groups, whereas serotypes 3 and 19A (both VT13) grew sharply, particularly in adults (Fig. 7B). Serotype growth between children and adults tended to be similar, with differences being mostly non-significant. Serotype 19A decreased much more in children than adults pre-PCV, whereas serotype 3 was growing more in adults than children. Despite being common in both age groups post-vaccination, serotype 14 decreased just as sharply post-vaccination as it increased pre-PCV in children and adults. Common serotypes that were increasing in rate of incidence were serotypes 6C and 22F in adults. On the other hand, serotype 8, 23A and 23B were all growing in incidence but rare in both age groups. This was the case for serotype 11A in children as well, although this serotype was already common in adult IPD. Finally, despite 9N being a highly ranked serotype post-PCV in adults, the rate of incidence actually declined.

#### France

The only VT serotypes increasing in incidence pre-PCV in France were serotypes 1 and 19A (children) and 7F (both age groups), despite the top ten rankings for both age groups consisting only of VTs in this period (Fig. 7C, Fig. 5C). Post-PCV13, common serotypes that were also increasing significantly were serotypes 10A, 15A, 15B/C, 22F, 23B, and 24F in children and serotypes 8, 9N and 15A in adults. Common serotypes that were not increasing significantly were serotypes 1 (children), 3, 6C, 19F, and 22F, (adults), and 7F, 12F, and 19A (both age groups). Serotypes that were rare (not included on the rank list, Fig. 5C) but increasing were serotypes 8 and 9N (children), and 10A, 16F, 23B (adults) and 11A, 23A, 35F, 38 (both age groups). Children and adults followed the same general trends of increase or decrease in incidence across serotypes.

#### Norway

Similar to other countries, VT7 serotypes were decreasing in frequency post-PCV7 in Norway in both age groups (Fig. 7D). Serotypes 7F and 19A were the only VT10/13 serotypes increasing after PCV7 in children and adults. Other serotypes increasing post-PCV7 were serotypes 24F (children), 10A, 22F and 23A (adults), and 6C and 23B (both age groups). Post-PCV13, common serotypes increasing in incidence were serotypes 24F (children), 8 and 9N (adults), and 22F (both age groups). Serotypes 1, 7F and 19A declined in incidence in adults, explaining the OR results from the previous section, and suggesting herd immunity. Common serotypes not increasing or decreasing significantly were 3, 7F, 23B, and 33F in both age groups. Surprisingly, serotypes 6A (VT13) and 19F (VT7) increased post-PCV in children. Rare serotypes increasing in incidence but not common were serotypes 8, 9N, 12F and 35F (children), and 10A, 12F, 15A, 16F, 24F and 35B (adults). Serotypes 8 and 9N were common in adults and 24F common in children.

#### USA

In the US, as expected, VT7 were all decreasing in incidence post-PCV7 in both age groups, whereas serotypes 7F and 19A were increasing (Fig. 7E). Other serotypes increasing significantly in incidence in this era were serotypes 15A, 23A and 23B (adults) and 6C (both age groups). Serotype 3 was the only serotype included in PCV13 that was increasing post-PCV13. Other serotypes whose incidence was significantly increasing post-PCV13 were serotype 38 (children), 15A and 23B (adults) and 35B (both age groups). VT10 and VT13 serotypes 7F and 19A, despite being common in both age groups, decreased in incidence. Unexpectedly, serotypes 6A (VT13) and 18C (VT7) increased post-PCV in children, although they were not common. The similarities between age groups suggests herd immunity from vaccination.

## Discussion

We have analysed serotype trends pre- and post-vaccination with PCVs in selected high-income countries in Europe, North America and Australia. These have high-quality surveillance data available that enables analysis of the serotypes causing the most IPD across settings and age groups, as well as allowing identification of serotypes that are growing in incidence post-vaccination. Following the introduction of expanded-valency vaccines, certain serotypes were common in all the settings included, resuting from the persistence of VT 19A and 3 and the emergence of NVTs 12F and 23B. However the serotype landscape between settings has diverged post-vaccination as a consequence of serotype replacement. As such, there are no specific dominating serotypes common to all countries, as was the case pre-vaccination with PCV7 or PCV10/13. We set out to compare the differences between countries relative to which vaccines were used. These comparisons may have been confounded by reporting inconsistencies between gathered national surveillance datasets. The USA data only listed the top 5 most common NVTs, and consequently the results of serotype-specific IPD as a percentage of total disease are overestimated (Fig. 6), results of NVT ORs are underestimated, and VT ORs overestimated (Fig. 1). Because it is the less abundant NVTs that are not reported, this should not alter the results significantly. Unfortunately, Finland was the only included country to introduce PCV10, and so systematic comparisons of PCV10 and PCV13 effects were not possible. Additionally, France and Australia were the only countries with pre-PCV7 data readily available, and there may have been other major serotypes in the pre-PCV7 era of other countries that were not captured. Although our aim was to analyse the serotype landscape in high-income settings, it is obviously important to compare the results reported in this analysis with lower income setting countries, where the IPD burden is highest. For instance, serotype 5 (VT10) is an important cause of IPD in Africa, but did not appear in any of the countries’ rankings lists before or after vaccination. This emphasizes both the need for high-quality surveillance data from lower income countries, and the challenges for designing vaccines for worldwide use.

We also aimed to assess patterns of relative abundance in IPD pre- and post-PCV. The OR results mirrored serotype replacement observations from previous reviews^17–19, 25–29^, indicating the proportion of IPD-causing NVT post-vaccination increased in all countries. The data suggest that herd immunity is also being established following the introduction of the higher-valency PCVs, although this process may not yet be complete. In Australia post-PCV13, VT10 were associated with a significantly greater proportion of disease relative to other serotype categories in adults aged 18–49 years, whereas they were associated with a significantly smaller proportion of disease relative to other serotype categories in children less than five. On the other hand, VT13 were associated with a smaller proportion of disease in both children less than 5 and all adults post-PCV13, suggesting herd immunity is being established. In Finland post-PCV10, the proportion of IPD caused by VT10 did not decrease significantly in children, but it was reduced in adults, which is likely a sign of herd immunity. The diversity analysis showed that while adult IPD was always caused by a relatively wide variety of serotypes, the serotypes causing IPD in infants became increasingly diverse after vaccination. The results are consistent with the rank frequency distributions where pre-PCV, few serotypes (i.e. VT7) were causing a majority of disease in children: in particular serotype 14 pre-PCV in Australia and Finland, and 19A pre-PCV13 in Australia, France, and the US. However, this was not the case in adults pre-PCV, nor in infants in Norway. This absence of dominating serotypes is now the situation for both infants and adults post-PCV10/13, as children’s and adults’ SDI became similar in most countries. However, Norway remains an exception, as the top-ranked serotype (22F) caused 18% of adult IPD. As the child serotype populations become more diverse, it becomes harder to achieve substantial IPD decreases with small increases in PCV valency.

Notably, serotype 3 was also consistently high in some countries after vaccination with PCV13. This may be attributable to a lower immunogenicity in the vaccine against this serotype, or this serotype’s relatively high propensity to be carried in older individuals, who are unvaccinated^30^. Targeting this serotype more effectively is important given its ubiquity across the countries in this study. It is not possible to quantitatively compare the benefit of targeting this serotype more effectively over expanding valency without accounting for any associated replacement, which would be expected to be small given the low colonisation rates^31^. However, the unusual properties of serotype 3’s age distribution could complicate the prediction of replacement effects in older adults. Unfortunately, even if this serotype was removed from circulation, there would still be a diverse range of serotypes to target in each country.

Comparing serotype growth and decline in age groups, one of the trends that emerged was that certain serotypes were common in one age group but rare and growing in incidence the other age group. Examples of this are seen in Finland (serotype 11A, common in adults, is increasing in children), France (serotypes 8 and 9N, common in adults, are increasing in children; serotypes 10A and 23B, common in children, are increasing in adults), and Norway (serotype 24F, common in children, is increasing in adults; serotypes 8 and 9N, common in adults, are increasing in children). Although it is unclear from the disease data whether adults may be a source of transmission in the community, the results reflect the known transmission between the two age groups, but may also suggest that serotype replacement is not yet complete. However, this is unclear without modelling the transmission and invasiveness of serotypes across age groups, which together would clarify whether these low-frequency but growing disease-causing serotypes are a result of replacement or age-specific invasiveness. Unfortunately, data on age-specific invasiveness are challenging to obtain at present as the carriage patterns in adults are not well understood, although additional data on adult IPD relative to infant carriage would be helpful in avoiding some of the adverse effects of paediatric immunisation programs on adult invasive disease.

The analysis of temporal changes in serotype abundance highlighted certain NVTs that appeared to be increasing significantly in multiple countries, such as serotypes 8, 9N, 15A, 23B in both young and older age groups. It is notable that no serotype has yet dominated IPD post-PCV in all countries as did serotype 14 pre-PCV and serotype 19A post-PCV7/10. There were some cases of NVTs decreasing without direct vaccination pressure, such as serotype 15A in adults in France, Norway and the USA. This may be a result of poorly understood ecological or serotype competition effects that are not explored in this analysis.

Finally, we aimed to assess the expansion of replacing NVT serotypes compared to those targeted by the vaccine, and identify potential candidates for serotypes to be included in new PCVs. Taken altogether, the odds ratio, Simpson’s Diversity Index and rank frequency results demonstrate that the replacing NVT serotypes are a diverse set, particularly in children where the loss of a dominating serotype in most locations post-vaccination is reflected in the increased diversity index. While vaccines tend to be implemented globally, the pooled SDI analysis for Australia, France and Norway indicates that there may be diminishing returns for adding more serotypes to new vaccines, as they will likely be proportionately less effective at reducing global IPD. This less skewed serotype distribution across IPD cases, described in our results and those reported elsewhere^32, 33^, is exacerbated by the post-PCV differences between countries and age groups. This makes it harder to tackle the remaining burden of disease with additional valency compared to previous PCVs. To complicate matters further, SDI, and therefore population structure, appears to be more similar between equivalent age groups of different countries. In contrast, the common serotypes appear to be more similar within countries across age groups. Hence each additional serotype added to PCVs can contribute only a relatively small further reduction in infant IPD, even in the absence of serotype replacement, and may only make such a contribution in a limited number of settings. This raises the possibility of requiring different vaccine serotype formulations for different locations, in line with ideas presented in a previous study^34^, as well as a distinct vaccine serotype formulations targeting adult IPD to prevent the replacement that is expected when expanding PCVs targeting infant carriage^34^. These factors make future universal PCV development and manufacturing challenging. Furthermore, despite having explored the trends of serotype replacement in disease, serotype epidemiology and temporal dynamics are greatly influenced by carriage, which is more difficult and costly to record. Understanding the rate at which certain serotypes cause disease given carriage may help in future vaccine design and in models predicting future serotype disease trends in various settings. For this, it is vital for public health bodies to continue recording surveillance data post-vaccination, and make this information publicly available, to understand both whether serotype replacement is complete post-PCV, and to facilitate comparisons in trends between countries. Ultimately, although formulations could in principle vary between locations (despite this likely complicating vaccine manufacturing), optimizing PCVs for both resource rich and resource poor countries promises to be challenging.

## Conclusion

Increasing vaccine valency may be increasingly difficult for vaccine manufacturers given the observed diversity of the prevalent serotypes between countries. Our analyses show that NVT serotypes 8, 9N, 15A and 23B are increasing in Australia, North America and certain European countries, even though their incidence trends are not consistent, and there are a variety of other NVTs affecting each country and age group. Additionally, although there are common emerging serotypes between countries and within age groups, there has not been a dominating serotype post-PCV as there was pre-PCV (serotype 14) and pre-PCV10/13 (serotype 19A). Public health bodies should continue to record surveillance data on serotype distribution, to facilitate both the prediction of future serotype trends and the optimization of the next generation of vaccine formulations.

## Methods

High income countries (as defined by the World Bank^35^) in Europe, North America and including Australia, with populations greater than 5 million people were chosen if they had a high PCV coverage (> 80%) and serotype-specific IPD national surveillance data publicly available from public health agencies, or published articles both before and after PCV vaccination (Supplementary Fig. 1), for all age groups, regardless of valency, vaccination schedule or number of surveillance years. Serotypes that were not typed or unknown were removed from the analyses.

### Statistical analyses

Statistical analyses were undertaken using R. An odds ratio (OR) and its 95% confidence interval (CI) were calculated for each vaccination era and age group to assess the association between vaccines implemented and the incidence of IPD caused by different serotype categories, comparing each vaccine and serotype category to the combination of all others. Serotype categories were vaccine types (VT) VT7 (PCV7 serotypes: 4, 6B, 9V, 14, 18C, 19F, and 23F), VT10 (additional PCV10 serotypes: 1, 5, 7F) and VT13 (additional PCV13 serotypes: 3, 6A, 19A), and non-vaccine serotypes (NVT). The purpose of the OR was to demonstrate how the proportion of VT7, VT10, VT13 or NVT-caused disease cases changed before and after vaccination in each age group. Our aim was to answer whether vaccines affected the serotype categories in the same way in each age category and country. Since countries have implemented different vaccines, the odds ratio estimated the proportion of these serotype categories after vaccination with whichever PCVs were implemented. The OR also circumvents the issue of calculating age-specific incidences when surveillance populations are aggregated from different sources, which can be complicated by non-vaccine effects on overall IPD incidence.

Simpson’s Diversity Index (SDI)^36^ was calculated to assess the diversity of the serotypes causing IPD each year for each country and age group, with confidence intervals^36^. SDI is an indication of richness and evenness of a population, considering the relative abundance of each species, and ranges from 0 (no diversity) to 1 (infinite diversity). We also estimated SDI for pooled countries 4 years post-vaccination, to understand whether vaccination was associated with divergence between countries’ IPD serotype compositions.

In order to understand whether individual serotypes had a strong influence on the OR and SDI results in each country, a rank frequency distribution for each vaccination era and country was plotted, with serotypes ranked according to the number of IPD cases, as well as a cumulative frequency curve, indicating a serotype's contribution to the total disease burden. Aggregation of disease cases per vaccination era allowed us to understand which serotype caused the most disease during different periods, making it insensitive to changes between individual years. These plots highlighted dominating serotypes, which we defined as the highest-ranked serotype(s) causing at least twice the burden of disease as the next most highly-ranked serotype. While the categories provide an overview of the vaccines’ impacts, each serotype within the categories may behave differently as well. For this reason, visualizing each individual serotype provided a more granular outlook that may elucidate the detailed patterns of serotype replacement.

To that end, the top 10 IPD-causing serotypes in each country at each timepoint were examined to compare between countries and age groups. The proportional contribution of these major serotypes were estimated as the disease cases caused by the individual serotype over total disease cases in order to compare the disease contribution between countries. A linear regression model was employed to obtain an estimate of the rate at which serotype incidence was growing or declining in each vaccination period for each country in order to understand which serotypes were growing or declining, and p-values were adjusted for multiple comparisons using the Benjamini and Hochberg method^37^. Serotypes that were both ranked in the top 10 and also had a high incidence growth rate were considered important for possible future inclusion in increased valency vaccines.

## Supplementary information

Supplementary Information.

## Data Availability

Data were publicly available from Finland’s National Institute for Health and Welfare (THL) and France’s Centre National de References des Pneumocoques (CNRP). Data from Norway and Australia were requested from Meldesystem for Smittsomme Sykdommer (MSIS) and National Notifiable Disease Surveillance System (NNDSS) respectively. Data from the United States were obtained from a published article^21^.
