# Supplementary material for: Divergent serotype replacement trends and increasing diversity in pneumococcal disease in high income settings reduce the benefit of expanding vaccine valency
Source: Sci Rep. 2020 Nov 4;10:18977. doi: 10.1038/s41598-020-75691-5 (PMC7643077; doi:10.1038/s41598-020-75691-5)
Supplement: Supplementary file 1 — Supplementary Information. [file 41598_2020_75691_MOESM1_ESM.pdf]

Divergent serotype replacement trends and increasing diversity in pneumococcal disease in high income settings reduce the benefit of expanding vaccine valency: Supplementary Material

Alessandra Lochen[1], Nick Croucher[1,2], Roy Anderson[1]

[1]Department of Infectious Disease Epidemiology, St Mary's Campus, Faculty of Medicine, Imperial College London, London W2 1PG

[2]Corresponding author: n.croucher@imperial.ac.uk, MRC Centre for Global Infectious Disease Analysis, Norfolk Place, Imperial College London, London W2 1PG, United Kingdom

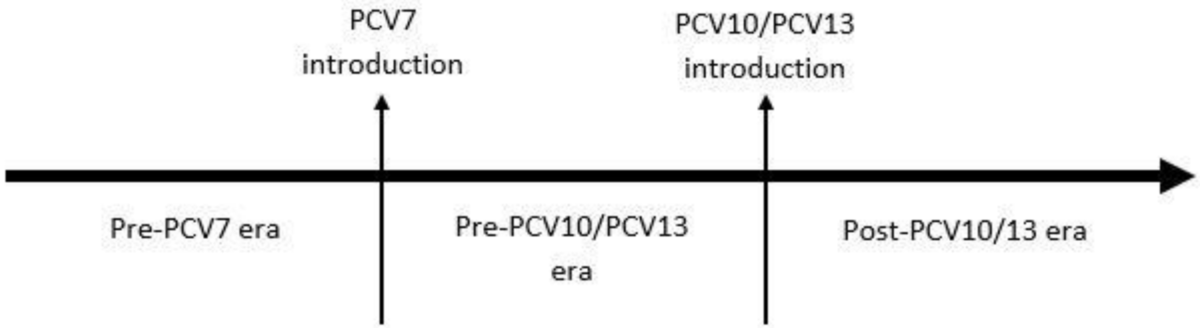

Supplementary Figure 1: Timeline of PCV implementation eras.

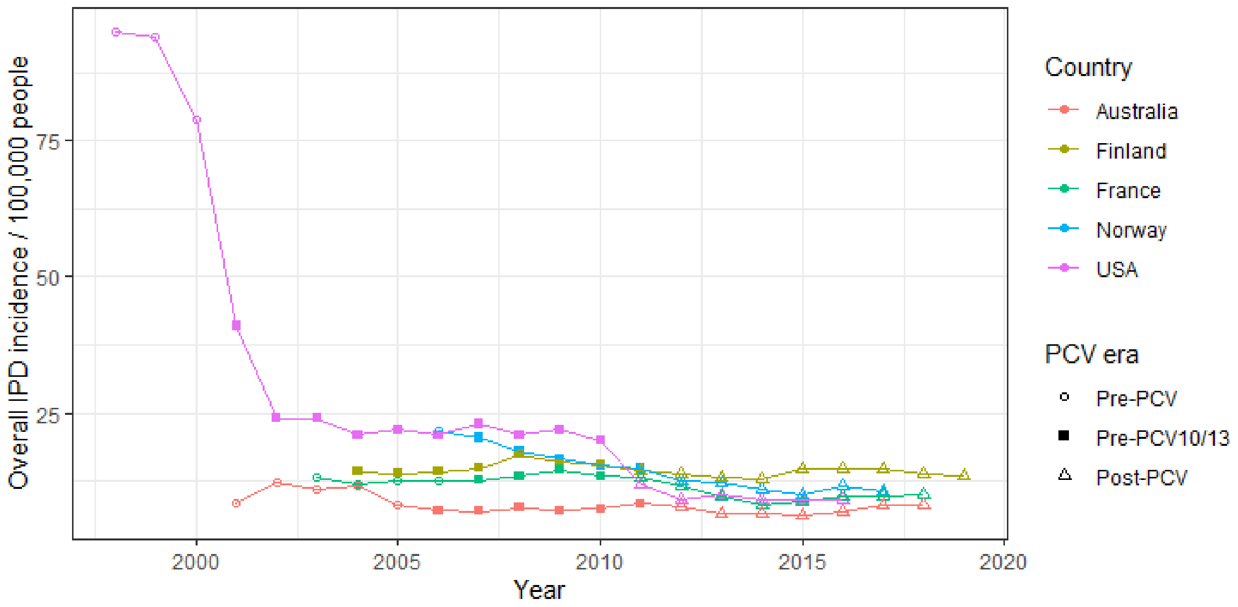

Supplementary Figure 2: National incidence of IPD per 100,000 people in Australia, Finland, France, Norway and USA pre- and post-vaccination with PCV7 and PCV10/13.

Supplementary Figure 3A: Comparison of incidence growth rates of VT in children between countries

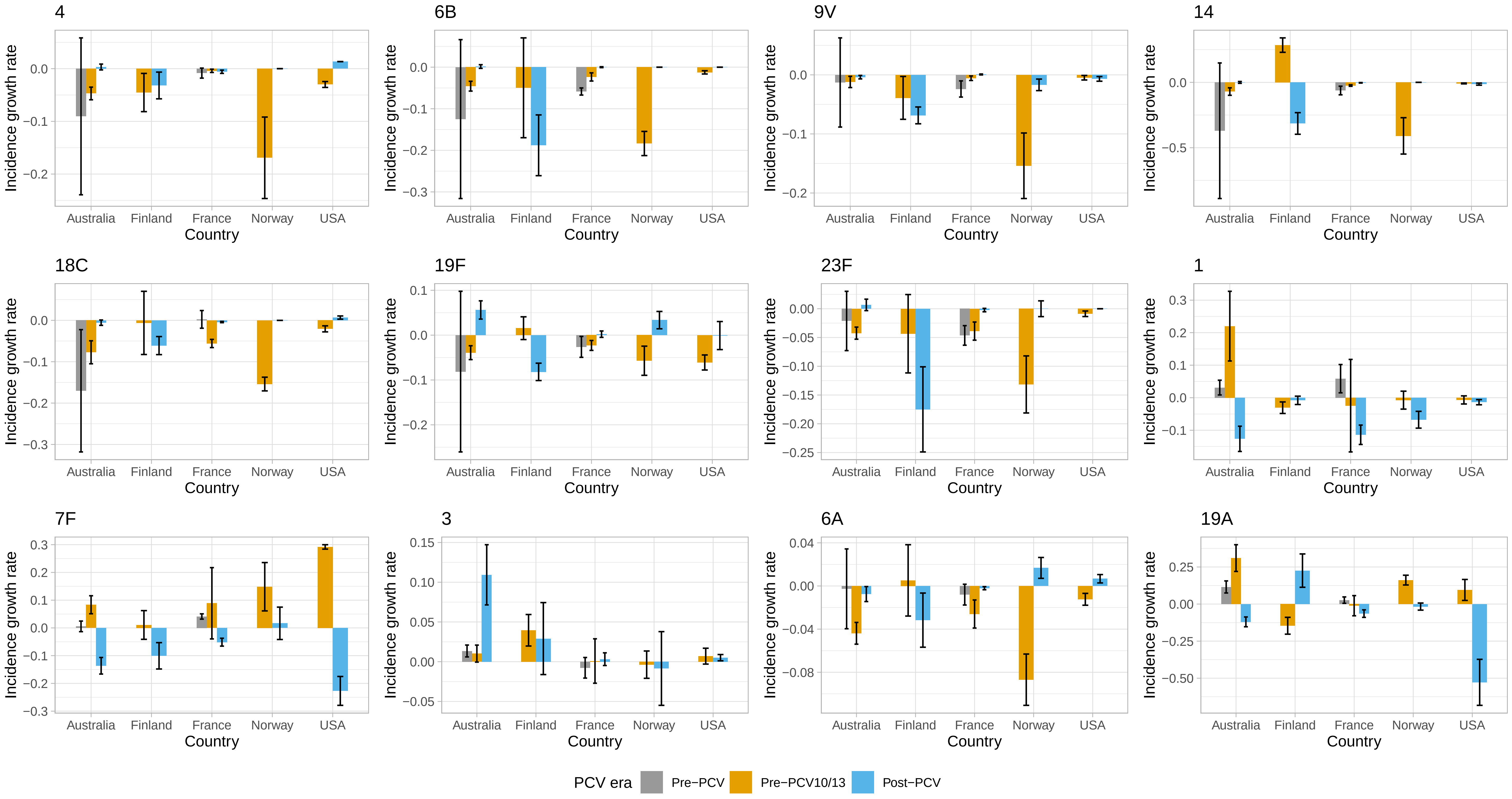
4
6B
9V
14
18C
19F
23F
1
7F
3
6A
19A
Incidence growth rate
Australia
Finland
France
Norway
USA
Country
PCV era
Pre−PCV
Pre−PCV10/13
Post−PCV

Supplementary Figure 3B: Comparison of incidence growth rates of NVT in children between countries

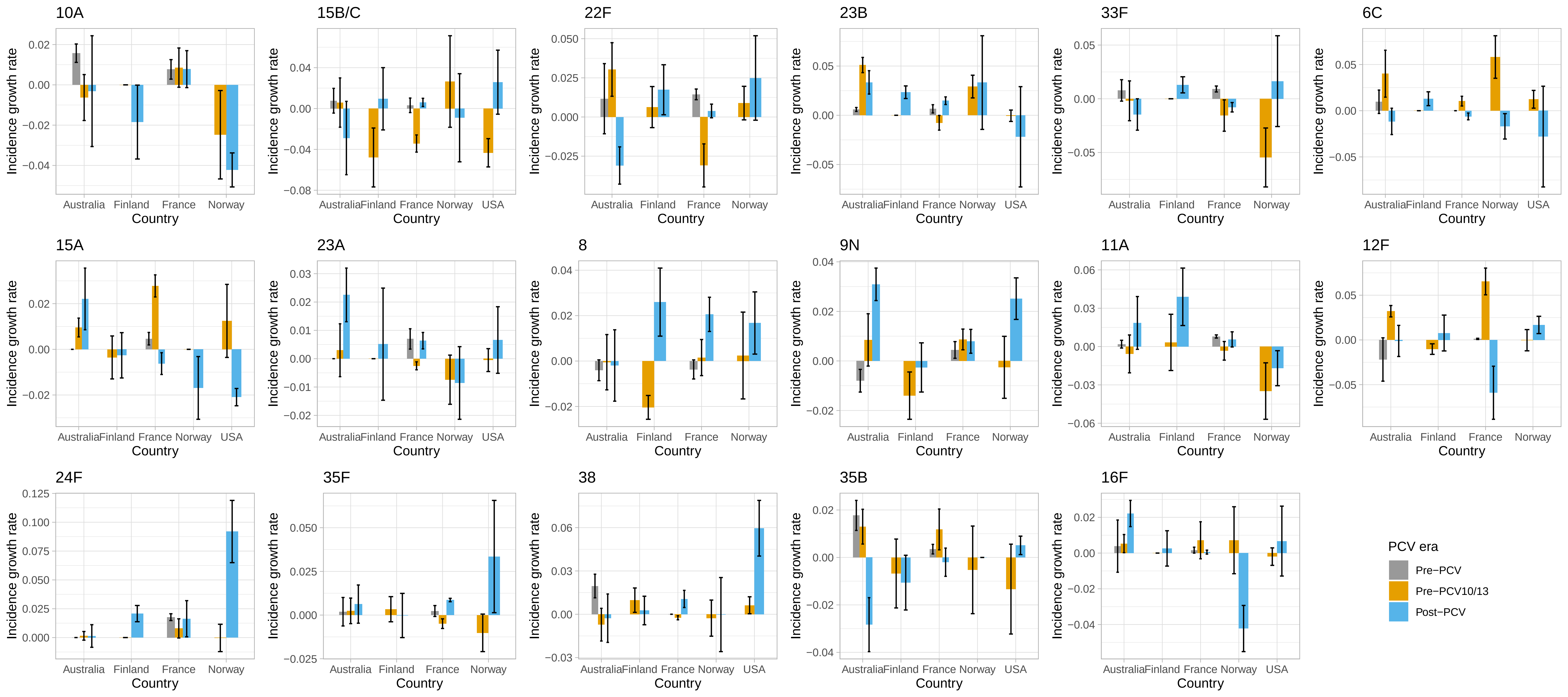
10A
15B/C
22F
23B
33F
6C
15A
23A
8
9N
11A
12F
24F
35F
38
35B
16F
Incidence growth rate
Country
Australia
Finland
France
Norway
USA
PCV era
Pre−PCV
Pre−PCV10/13
Post−PCV

Supplementary Figure 3C: Comparison of incidence growth rates of VT in adults between countries

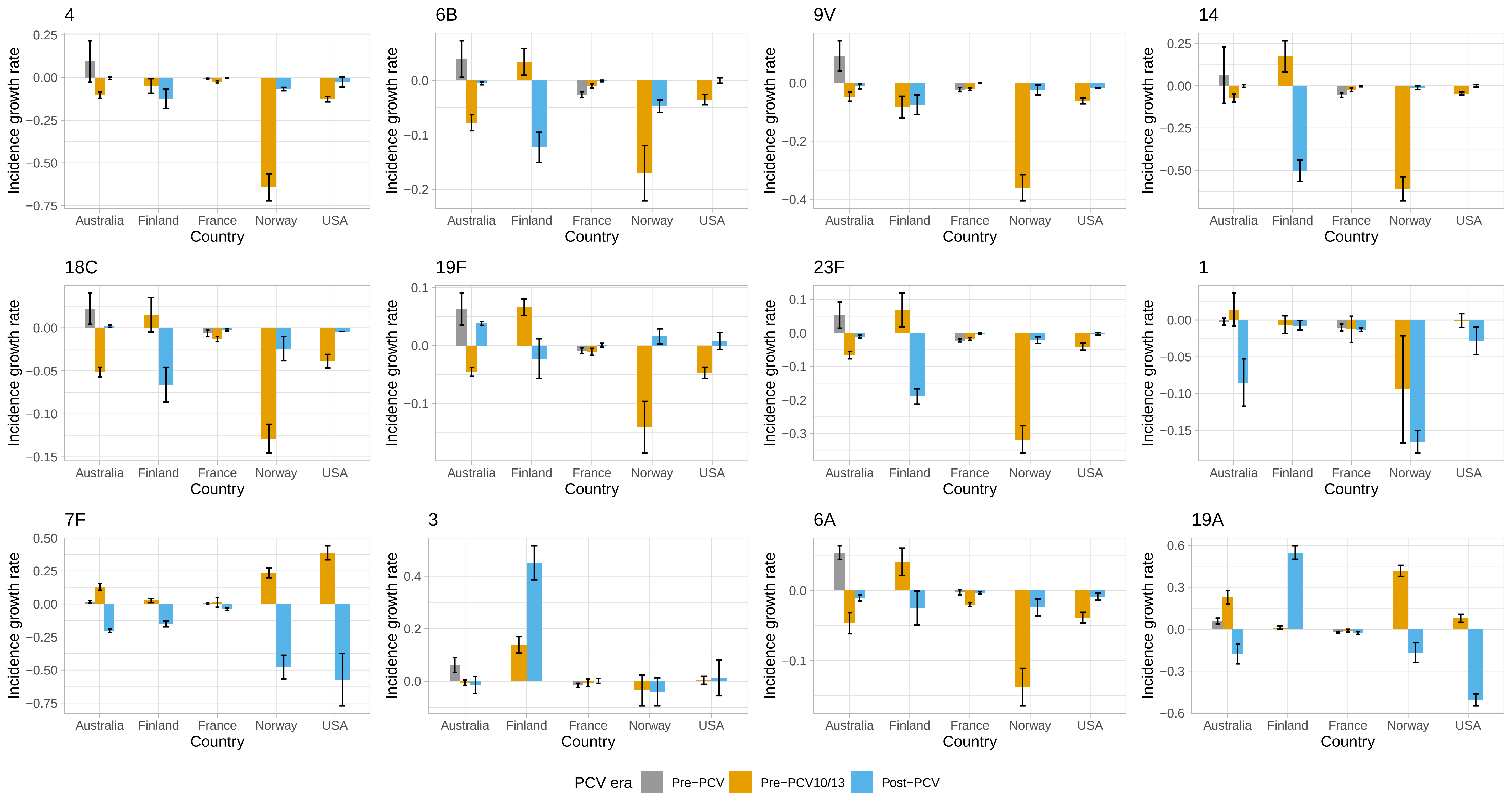
4
6B
9V
14
18C
19F
23F
1
7F
3
6A
19A
Incidence growth rate
Country
Australia
Finland
France
Norway
USA
PCV era
Pre−PCV
Pre−PCV10/13
Post−PCV

Supplementary Figure 3D: Comparison of incidence growth rates of NVT in adults between countries

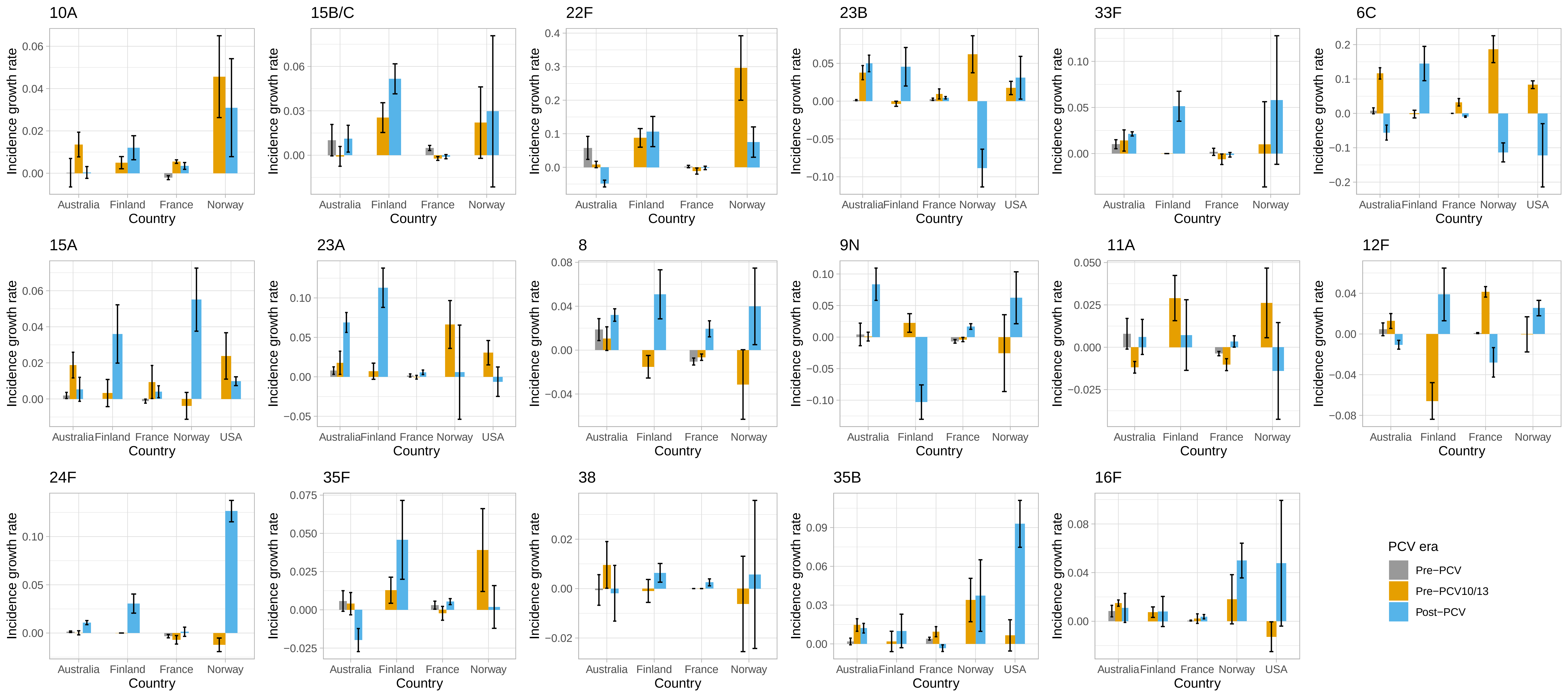
10A
15B/C
22F
23B
33F
6C
15A
23A
8
9N
11A
12F
24F
35F
38
35B
16F
Incidence growth rate
Country
Australia
Finland
France
Norway
USA
PCV era
Pre−PCV
Pre−PCV10/13
Post−PCV

Supplementary Figure 4A: Incidence of VTs in children in different countries

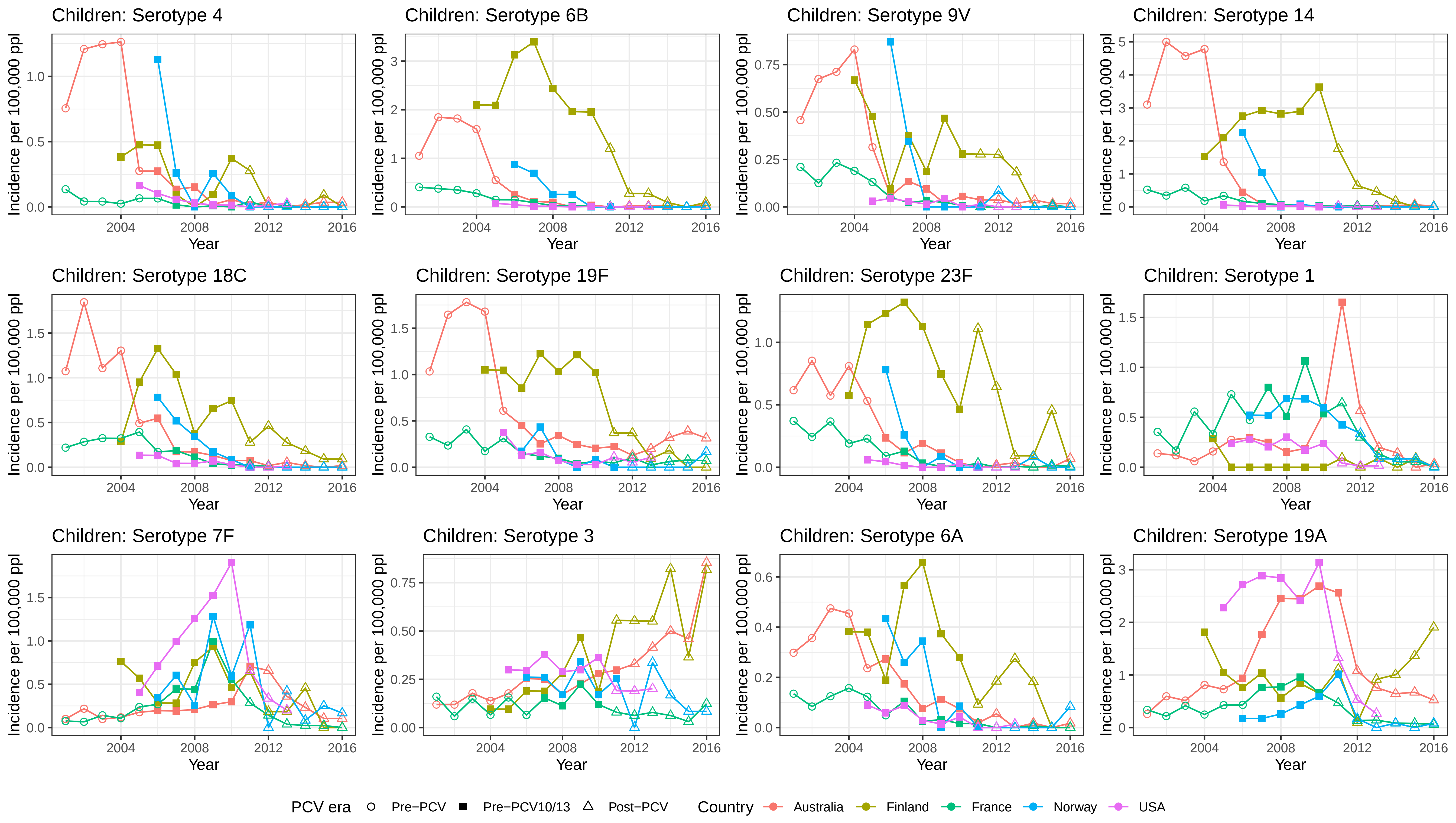

Children: Serotype 4
Children: Serotype 6B
Children: Serotype 9V
Children: Serotype 14
Children: Serotype 18C
Children: Serotype 19F
Children: Serotype 23F
Children: Serotype 1
Children: Serotype 7F
Children: Serotype 3
Children: Serotype 6A
Children: Serotype 19A
Incidence per 100,000 ppl
Year
PCV era
Pre−PCV
Pre−PCV10/13
Post−PCV
Country
Australia
Finland
France
Norway
USA

Supplementary Figure 4B: Incidence of NVTs in children in different countries

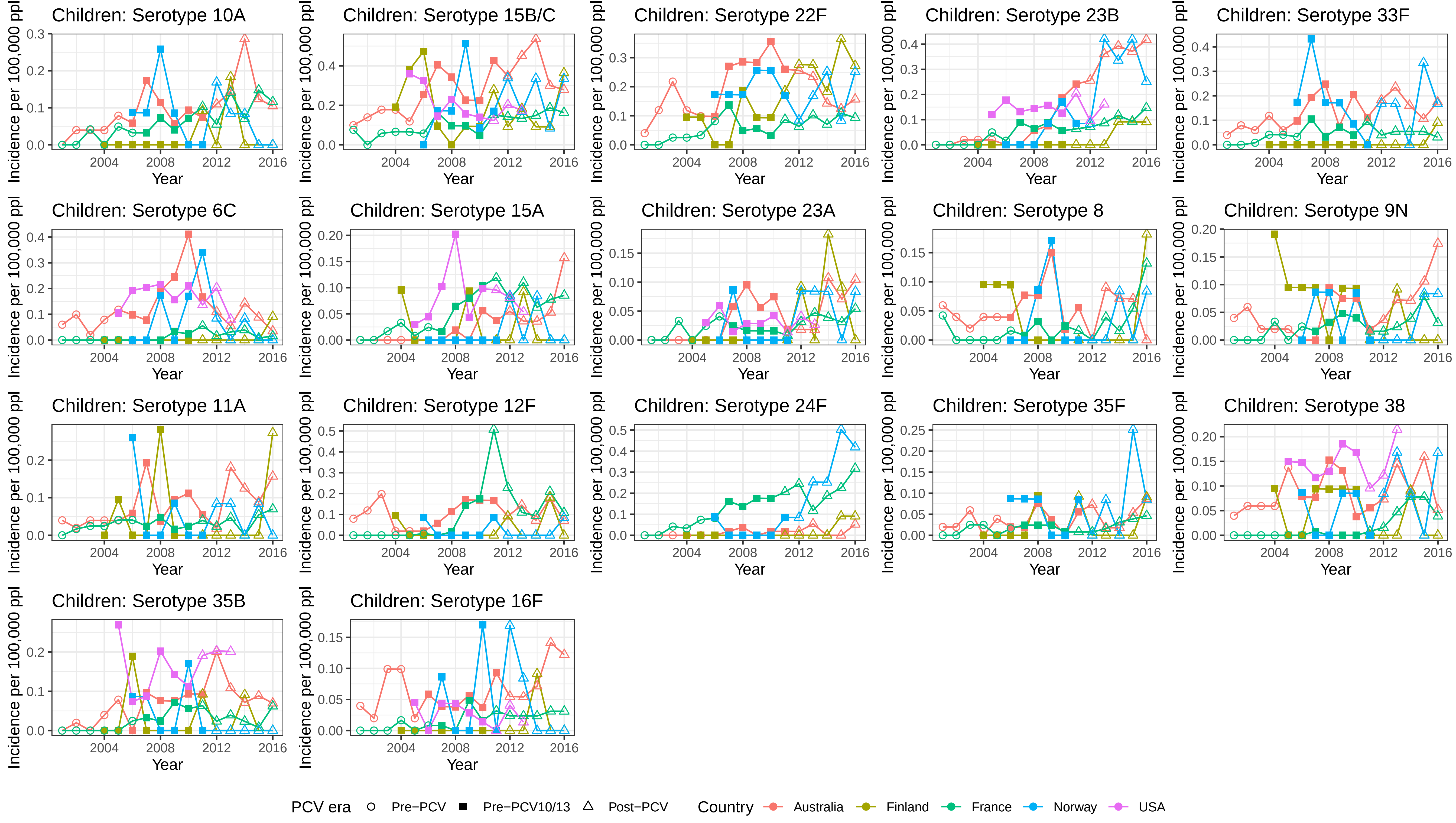
Children: Serotype 10A
Children: Serotype 15B/C
Children: Serotype 22F
Children: Serotype 23B
Children: Serotype 33F
Children: Serotype 6C
Children: Serotype 15A
Children: Serotype 23A
Children: Serotype 8
Children: Serotype 9N
Children: Serotype 11A
Children: Serotype 12F
Children: Serotype 24F
Children: Serotype 35F
Children: Serotype 38
Children: Serotype 35B
Children: Serotype 16F
Incidence per 100,000 ppl
Year
PCV era
Pre−PCV
Pre−PCV10/13
Post−PCV
Country
Australia
Finland
France
Norway
USA

Supplementary Figure 4C: Incidence of VTs in adults in different countries

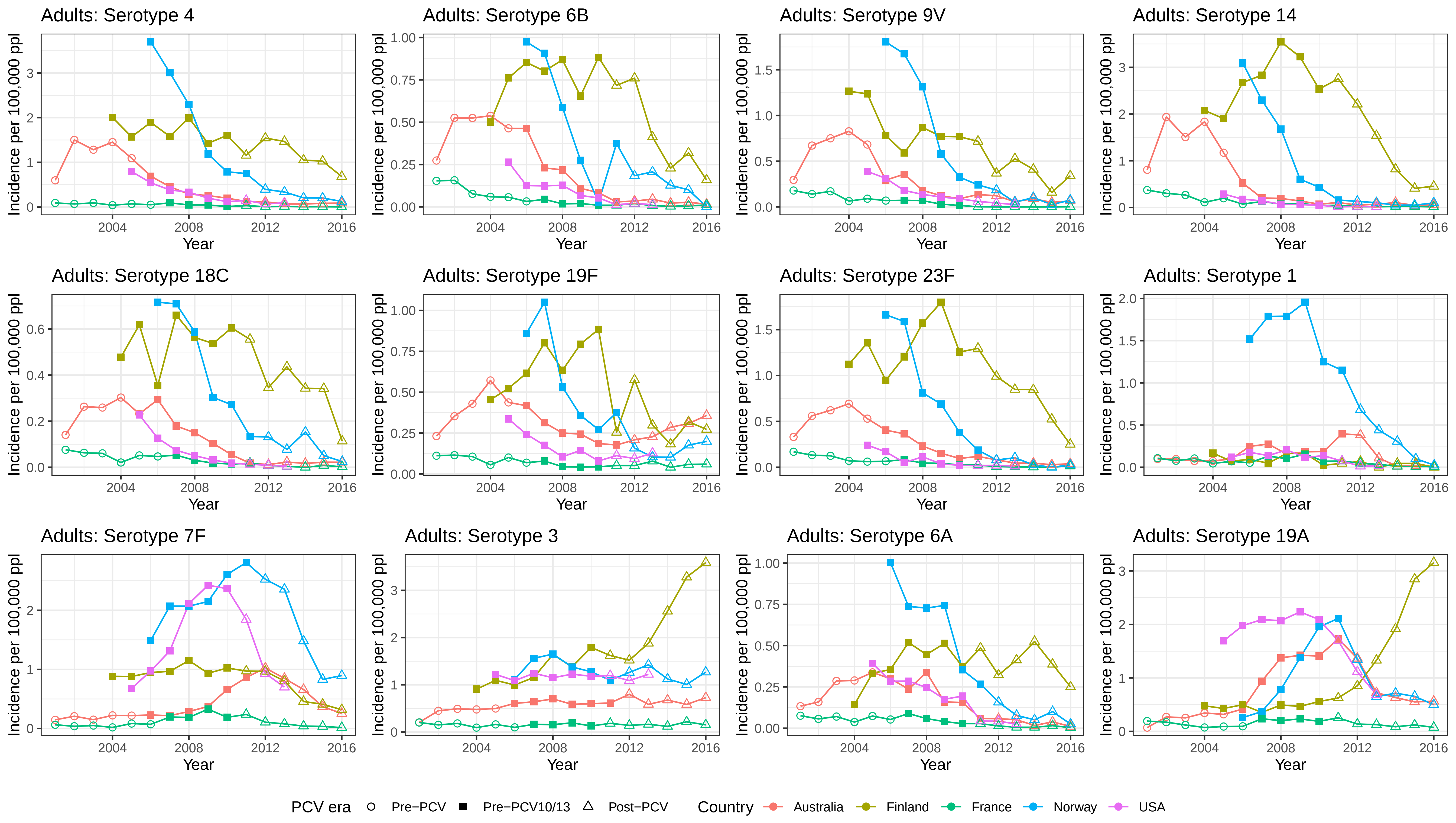

Adults: Serotype 4
Adults: Serotype 6B
Adults: Serotype 9V
Adults: Serotype 14
Adults: Serotype 18C
Adults: Serotype 19F
Adults: Serotype 23F
Adults: Serotype 1
Adults: Serotype 7F
Adults: Serotype 3
Adults: Serotype 6A
Adults: Serotype 19A
Incidence per 100,000 ppl
Year
PCV era
Pre−PCV
Pre−PCV10/13
Post−PCV
Country
Australia
Finland
France
Norway
USA

Supplementary Figure 4D: Incidence of NVTs in adults in different countries

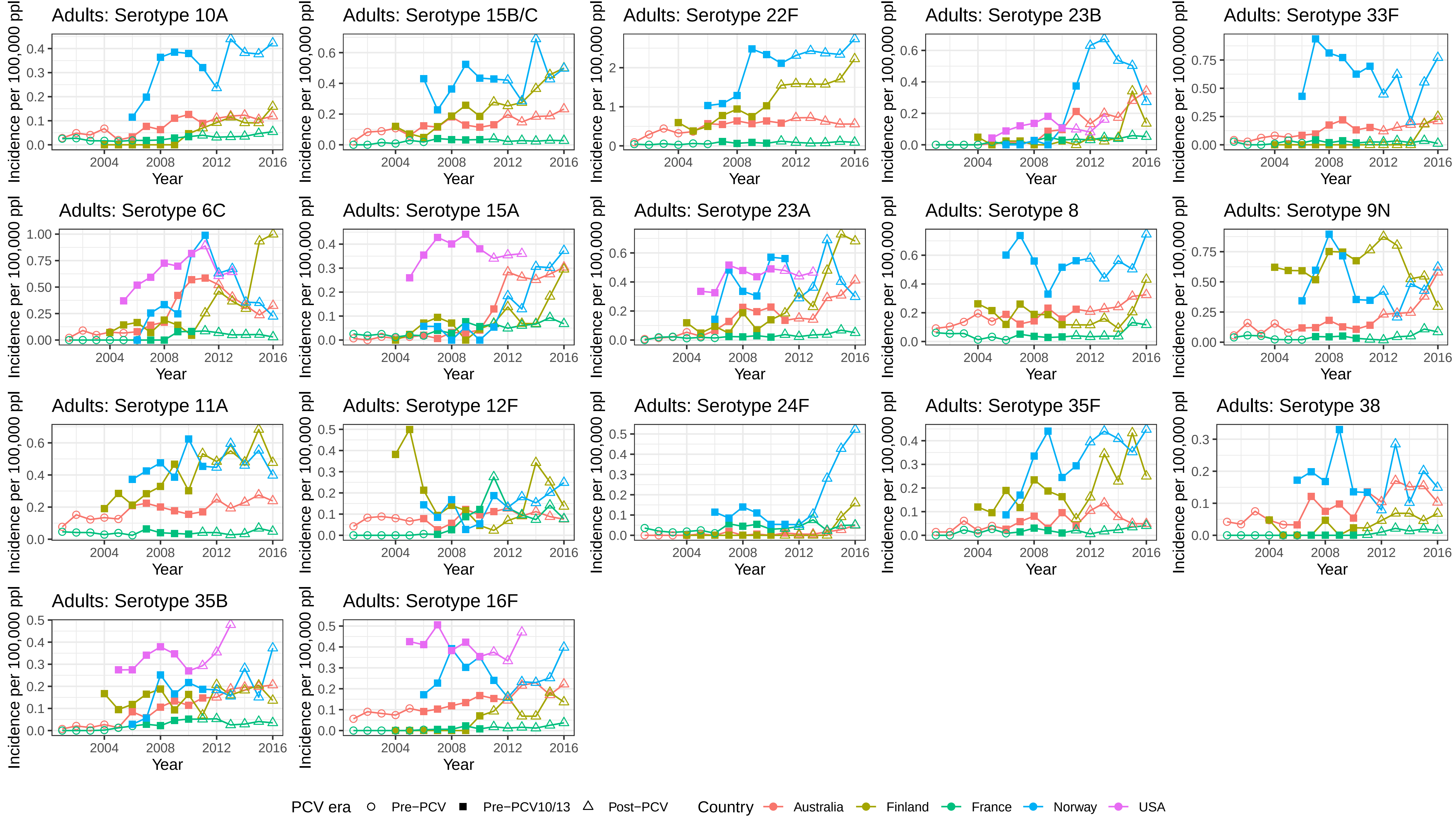
Adults: Serotype 10A
Adults: Serotype 15B/C
Adults: Serotype 22F
Adults: Serotype 23B
Adults: Serotype 33F
Adults: Serotype 6C
Adults: Serotype 15A
Adults: Serotype 23A
Adults: Serotype 8
Adults: Serotype 9N
Adults: Serotype 11A
Adults: Serotype 12F
Adults: Serotype 24F
Adults: Serotype 35F
Adults: Serotype 38
Adults: Serotype 35B
Adults: Serotype 16F
Incidence per 100,000 ppl
Year
PCV era
Pre−PCV
Pre−PCV10/13
Post−PCV
Country
Australia
Finland
France
Norway
USA
